# Physicochemical, microbial, and sensory characteristics of yogurt with Persian shallot (*Allium hirtifolium* Boiss) and probiotic bacteria

**DOI:** 10.1002/fsn3.4036

**Published:** 2024-02-13

**Authors:** Farahnaz Vahdat, Tooraj Mehdizadeh, Hamidreza Kazemeini, Anna Reale, Ata Kaboudari

**Affiliations:** ^1^ Department of Food Hygiene, Faculty of Veterinary Medicine Amol University of Special Modern Technologies Amol Iran; ^2^ Department of Food Hygiene and Quality Control Urmia University Urmia Iran; ^3^ Institute of Food Science National Research Council (ISA‐CNR) Avellino Italy

**Keywords:** *Bifidobacterium bifidum*, *Lactobacillus acidophilus*, natural additives, synbiotic

## Abstract

The aim of this study was to investigate the characteristics of yogurt prepared with the addition of Persian shallot and probiotic bacteria. The effect of Persian shallot on the viability of probiotic bacteria (*Lactobacillus acidophilus* and *Bifidobacterium bifidum*) was evaluated. Furthermore, the antimicrobial effects of shallot and probiotic bacteria on *Listeria monocytogenes* and *Escherichia coli* species were investigated. The experiments were performed on days 0, 1, 7, 14, and 21. The results showed that the survival of lactic acid bacteria increased significantly in the presence of shallots (*p* < .05). The addition of two different probiotic bacteria to the yogurt samples inhibited the pathogenic bacteria. While *E. coli* bacteria had a 3‐log reduction, *L. monocytogenes* did not grow at all in the presence of probiotic bacteria and shallots. Based on these experiments, it was concluded that the addition of shallots not only increased the survival of probiotic bacteria but also reduced the growth of food‐borne pathogenic bacteria. In addition, the addition of probiotic bacteria increased the acceptance of sensory properties of yogurt samples.

## Graphical abstract

The aim of this study was to investigate the characteristics of a synbiotic yogurt enriched by Persian shallots and probiotic bacteria. The effect of shallots on probiotic bacteria viability was evaluated. Furthermore, the antimicrobial effects of shallots and probiotic bacteria on *Listeria monocytogenes* and *Escherichia coli* species were investigated.

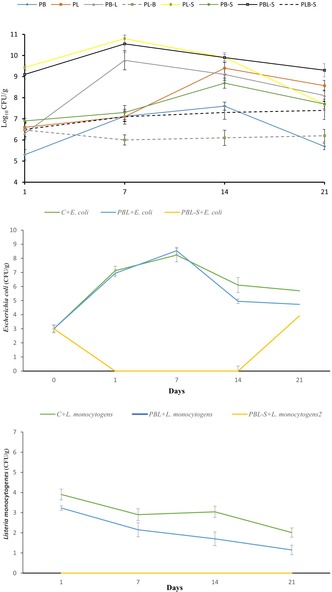

## 1 INTRODUCTION

Dairy products are one of the most important components of human nutrition (FAO/WHO, 2002). Due to the amount of energy, proteins, and fats, and especially vitamins and minerals, dairy products play an essential role in nourishing human communities (Granato et al., 2018). Among the many types of dairy products, yogurt holds a special place, as it is widely regarded as a healthy product and is used to deliver probiotics and prebiotics to consumers. However, not all yogurts are probiotics nor are all of them functional products (Chugh & Kamal‐Eldin, 2020; Nyanzi et al., 2021).

One of the most important factors in ensuring the probiotic effect of yogurts is the survival rate of probiotic bacteria that must survive within yogurt throughout its entire shelf‐life and within the gastrointestinal tract after consumption to provide health benefits (Mehdizadeh et al., 2021; Olson & Aryana, 2022). *Bifidobacteria* and *Lactobacilli* are the most common groups of bacteria that are added to foods for their probiotic properties (Mehdizadeh et al., 2019). Prebiotics, on the other hand, are ingredients that stimulate the growth of healthy bacteria. Prebiotics are found in many fruits and vegetables such as leek, chicory, wheat, asparagus, oats, garlic, soybean, artichoke, and many other plants (Garcia‐Alonso et al., 2022).

The survival of probiotic bacteria as well as the presence of prebiotic substances in dairy products is considered a major challenge in the functional food industry (Ozcan et al., 2021; Reale et al., 2019; Savaiano & Hutkins, 2021).

One of the strategies studied by researchers in recent years to increase the growth and survival of probiotics in food products during storage and in the gut after consumption is the use of probiotics and prebiotics in the form of synbiotic products (Kariyawasam et al., 2021; Xu et al., 2023). Synbiotic products, in which a combination of probiotics and prebiotics are used, improve the survival and selective implantation of useful live microbial strains in the digestive system. Synbiotic products have numerous health benefits such as antimicrobial, anticancer, antiallergic, and immune system stimulating (Ban et al., 2020; Fazilah et al., 2018). Today, researchers are interested in innovative processes such as enriching functional yogurt products with probiotics, prebiotics, or a combination of them. Yogurt is one of the fermented dairy foods that is a suitable environment for the growth of all kinds of microorganisms despite the acidic conditions (Cutrim et al., 2016). Therefore, the control of foodborne pathogens (such as *Escherichia coli*) in this product is one of the attractive topics for development in this field (Kamal et al., 2018).

In recent years, many scientists have incorporated probiotic and plant‐derived prebiotics into dairy products, especially in yogurt (Aliza Sigdel et al., 2018; Azizkhani & Tooryan, 2016; Fathy et al., 2022; Fazilah et al., 2018; Hashemi & Hosseini, 2021; Khalid et al., 2022; Pereira et al., 2021; Ranadheera et al., 2012; Sánchez‐Vega, 2013; Sohrabpour et al., 2021; Turgut & Cakmakci, 2018).

Shallots, for example, added to a variety of foods such as salads, pickles, and different sauces (Harris et al., 2001) are also added to yogurt in some eastern countries.

The Persian shallot (*Allium hirtifolium* Boiss) is one of the most widely used medicinal plants in Iran, its main habitat being the Zagros Mountains in western and central Iran. This plant belongs to the genus Allium and the family Alliacea. Allium species, such as garlic and onion, have been used to treat various diseases (Mohammadiani et al., 2021). Antibacterial, antifungal, antitumor, and anthelmintic effects have been demonstrated for many members of this genus. In addition, several types of shallots contain sugars and inulin‐type polysaccharides, which are considered an excellent prebiotic with beneficial effects on human health (Major et al., 2022).

The aim of the present study was to evaluate the effect of Persian shallots on the quality characteristics of probiotic yogurt, also assessing the potential antimicrobial effect of Persian shallots on probiotic bacteria and the most common pathogenic microorganisms that might be present in yogurt.

## 2 MATERIALS AND METHODS

### 2.1 Persian shallots preparation

Persian shallot bulbs were purchased from the Grand Bazaar in Urmia and approved by the Department of Agriculture, Faculty of Agriculture, Urmia University. Fresh shallots, being root vegetables, were washed with drinking water and then peeled. Then, the shallots were placed in cold water for 24 h, replacing the water every hour, to eliminate the spicy flavor characteristic of fresh shallots. Afterward, they were spread on a clean cloth and dried at 25°C. The final moisture content was 17/5 ± 2%. After drying, they were cut to a size smaller than 1 mm before use.

### 2.2 Probiotic strains and culture conditions

*Lactobacillus acidophilus* (LA_5) (PTCC1608) and *Bifidobacterium bifidum* (BB_12) (PTCC1644), stored at the Bacterial and Fungal Collection Center of the Iranian Scientific and Industrial Research Organization, were used in the study.

The strains *L. acidophilus* and *B. bifidum* were reactivated and cultured, respectively, in the medium MRS broth (Sigma‐Aldrich, Germany) at 37°C and MRS modified (MRS broth with 0.05 g/100 g of L‐cysteine hydrochloride) at 37°C under anaerobic conditions (using Gazpack System) until exponential phase was reached. After incubation, the grown cells were harvested by centrifugation (4500 × *g*, 15 min, 4°C) and washed twice with sterilized peptone water solution (0.1 g 100 mL^−1^).

Each probiotic culture was subsequently added to milk for yogurt preparation at a concentration of about 1 × 10^8^ cfu mL^−1^ (Mehdizadeh et al., 2019).

### 2.3 Pathogen strains and culture conditions

*Listeria monocytogenes* (ATCC19115) and *Escherichia coli* O157:H7 (ATCC1533), stored at the Department of Food Hygiene and Quality Control, Faculty of Veterinary Medicine, Urmia University, Urmia, Iran, were used in the study. *E. coli* and *Listeria monocytogenes* were cultured in BHI medium (Merck, Darmstadt, Germany) at 37°C for 24 h. After incubation, the grown cells were harvested by centrifugation (4500 × *g*, 15 min, 4°C) and washed twice with a sterilized peptone water solution (0.1 g 100 mL^−1^) to be added later to milk for the yogurt preparation.

Each pathogenic strain was added to the milk for the yogurt preparation at a concentration of about 1 × 10^3^ cfu mL^−1^.

### 2.4 Yogurt production procedure

For yogurt preparation, fresh whole cow's milk (with 8.09% of SNF material) was pasteurized at 95°C for 5 min and cooled. About 14 g L^−1^ of shallot was added to the milk and it was portioned into sterile 500 mL containers. The milk temperature was raised to 41°C before inoculum. The yogurt starters, including *Lactobacillus delbrueckii* ssp. *bulgaricus* and *Streptococcus thermophilus* (Ch. Hansen, YC‐X11, Horsholm, Denmark) were added in a ratio of 1/1 at a concentration of 1 × 10^8^ cfu mL^−1^. Probiotic bacteria were added at a final concentration of about 1 × 10^8^ cfu mL^−1^. The samples were then incubated for 24 h at 41°C and then refrigerated at 4°C for 21 days. Microbiological and physicochemical analyses were performed at time 0 and after 1, 7, 14, and 21 days (Mehdizadeh et al., 2019). In some samples, pathogenic microorganisms were added as described in Section 2.3.

A total of 11 types of yogurt were prepared. These 11 treatments selected in this research were selected based on the presence or absence of Persian shallot and the type of added bacteria. The treatments include probiotic‐free and shallot‐free yogurt, starter only (Control sample), yogurt with shallot (CS), Bifidobacterium probiotic yogurt (PB), Lactobacillus probiotic yogurt (PL), Bifidobacterium and Lactobacillus probiotic yogurt (PB‐L), Bifidobacterium and shallot yogurt (PB‐S), Lactobacillus and shallot yogurt (PL‐S), Bifidobacterium and Lactobacillus with shallot yogurt (PBL‐S), Bifidobacterium and Lactobacillus probiotic yogurt containing shallot and *E. coli* (PBL‐S + *E. coli*), Bifidobacterium and Lactobacillus probiotic yogurt with shallot and *Listeria monocytogenes* (PBL‐S + *L. monocytogens*), and Bifidobacterium and Lactobacillus probiotic yogurt with *L. monocytogens* and *E. coli* without shallot (PBL + *L. monocytogens* and *E. coli*).

### 2.5 Physicochemical and chemical characteristics

Protein, fat, and solids not fat (SNF) were determined according to the methodology of the Association of Official Analytical Chemists (AOAC, 2012). The protein level was evaluated by the micro‐Kjeldahl procedure (Mehdizadeh et al., 2019). The fat obtained was dried to constant weight and determined as fat percentage by weight. Not fat solids (SNF) were evaluated by determining total solids and fat measures. The fat percentage was deducted from the total solids and used to calculate the SNF. The assays were carried out in triplicate and the results were expressed as mean values ± standard deviations.

To determine acidity, exactly 9 g of yogurt was poured into a beaker, and the equivalent of distilled water without carbon dioxide and 0.5 mL of 5% alcohol phenolphthalein reagent were added. The yogurt samples were then titrated. The titratable acidity was expressed in terms of % lactic acid (AOAC, 2012).

The pH was recorded using a Corning pHmeter (Hanna Instruments, Italy). To determine the pH of the yogurt, the pH meter was placed directly inside the yogurt samples and the pH was read.

### 2.6 Measurement of syneresis

Syneresis of the yogurt was assessed through the centrifugation procedure given by Soni et al. (2020) with some modifications. Briefly, 15 g of each sample was carefully weighed in each of the centrifuge falcons and, after closing the lid, was centrifuged at 3500 rpm (2058 × *g*) for 30 min at 4°C. The separated liquid was weighed and recorded as the percentage of syneresis.

### 2.7 Microbial analysis

For microbial analysis, 1 mL of yogurt sample was suspended in 9 mL of sterile peptone water (0.1%) and serial dilutions were made in the same medium before plating. Enumeration of *B. bifidum* was performed on MRS agar (Sigma‐Aldrich, Germany) with the addition of nalidixic acid (0.0015% w/v), neomycin sulfate (0.1% w/v), lithium chloride (0.3% w/v), and l‐cysteine hydrochloride (0.05% w/v) after incubation at 37°C for 72 h under anaerobic condition using a gas pack system (Merck Anaerocult Type A, Darmstadt, Germany). Enumeration of *L. acidophilus* was performed on MRS agar containing ciprofloxacin and clindamycin antibiotics as well as bile after anaerobic incubation at 37°C for 72 h.

Violet red bile lactose agar (VRBL) culture medium (Merck, Darmstadt, Germany) was used to count coliform bacteria after incubation at 37°C for 24 h. Yeast extract dextrose chloramphenicol agar medium at 25°C was used to evaluate and count mold and yeasts with incubation at 25°C for 5 days and 5°C for 10 days, respectively (Shahein et al., 2022). All analyses were performed in triplicate.

PALCAM agar medium (Oxoid, United States) was used to evaluate and count *Listeria monocytogenes*. The counts of *E. coli* were enumerated on MacConkey agar–sorbitol (Sigma‐Aldrich, Germany) and incubated at 35°C for 18–24 h (Al‐Nabulsi et al., 2016).

### 2.8 Sensory evaluation

Sensory analysis was carried out on the basis of a hedonic scale test (0–4) according to the method described by Mehdizadeh et al. (2019) with some modifications. In this method, 0 was the lowest evaluation score and 4 was the highest. In this sensory analysis, taste, texture, color, and general appearance on the last day of storage were examined. The conditions of sensory tests were explained to all participants. The analysis was performed only on the samples C, CS, PB, PL, PB‐L, PB‐S, PL‐S, and PBL‐S.

### 2.9 Statistical analysis

All experiments were done in triplicate and two samples were analyzed for each treatment. One‐way analysis of variance (ANOVA) was performed using statistical software (SPSS 24, Statistical Software Inc., Chicago, III). Duncan's test was used to compare means and significant differences between different treatments and combinations with confidence limits (*p* < .05).

## 3 RESULTS AND DISCUSSION

### 3.1 Physicochemical characteristics

The amounts of fat, protein, and SNF measured in the yogurt samples are shown in Table 1. The amount of fat was similar in all samples analyzed and ranged from 2.38 (sample PB) to 2.51 (sample PBL‐S), with no significant difference. Indeed, the amount of SNF and protein was significantly higher in the samples containing shallots than in those without. The samples with shallot had a protein content between 3.21 (CS) and 3.32 (PL‐S), whereas sample without shallot had a protein content of about 3.0. The samples with shallot had protein content between 3.21 (CS) and 3.32 (PL‐S), whereas sample without shallot had a protein content of about 3.0. The SNF values were around 8.0 in the samples without shallot and about 9.0 in the samples of yogurt produced with shallot. These differences are certainly due to the fact that Persian shallots contain a good source of protein, as well as being rich in vitamin C, potassium, fiber, folic acid, calcium, and iron which can have an effect on the growth of lactic acid bacteria (Moradi et al., 2013).

**TABLE 1:** Amounts of fat, protein, and SNF in the control and treated yogurt samples.

Parameter	Samples							
	C	CS	PB	PL	PB‐L	PB‐S	PL‐S	PBL‐S
Fat (%)	2.40 ± 0.18^a^	2.45 ± 0.06^a^	2.38 ± 0.11^a^	2.42 ± 0.08^a^	2.40 ± 0.19^a^	2.47 ± 0.21^a^	2.46 ± 0.19^a^	2.51 ± 0.16^a^
Protein (%)	3.01 ± 0.03^a^	3.21 ± 0.07^b^	3.03 ± 0.06^a^	3.04 ± 0.12^a^	3.06 ± 0.08^a^	3.29 ± 0.05^b^	3.32 ± 0.06^b^	3.31 ± 0.14^b^
SNF (%)	8.13 ± 0.09^a^	9.20 ± 0.15^b^	8.19 ± 0.11^a^	8.20 ± 0.13^a^	8.28 ± 0.09^a^	9.16 ± 0.06^b^	9.18 ± 0.13^b^	9.09 ± 0.05^b^

*Note*: Values are expressed as mean ± SD of three replicates. Lowercase letters (a, b) in the same row indicate significant differences (*p* < .05) among the eight different samples.

Abbreviations: C, Probiotic‐free and shallot‐free yogurt, starter only; CS, yogurt with shallots; PB, Bifidobacterium probiotic yogurt; PB‐L, Bifidobacterium and Lactobacillus probiotic yogurt; PBL‐S, Bifidobacterium and Lactobacillus with shallot yogurt; PB‐S, Bifidobacterium and shallot yogurt; PL, Lactobacillus probiotic yogurt; PL‐S, Lactobacillus and shallot yogurt.

The results of pH, titratable acidity, and syneresis of the probiotic yogurt samples during shelf life are shown in Table 2. The pH of the control C and CS samples were 4.0 and 4.67 on day 1, respectively, and then decreased over time during the 21‐day shelf‐life. In general, the yogurt produced with shallot had higher pH values than the sample produced without shallot. This effect is related to the buffering effect of shallots.

**TABLE 2:** pH, titratable acidity, and syneresis of the yogurt samples during 21 days of shelf‐life at 4°C.

Parameter	Treatment	Days			
		1	7	14	21
pH	C	4.0 ± 0.09^dA^	3.41 ± 0.06^bB^	3.40 ± 0.04^aB^	3.46 ± 0.03^aB^
	CS	4.67 ± 0.14^eA^	4.70 ± 0.1^fA^	4.63 ± 0.09^dA^	4.53 ± 0.11^dA^
	PB	3.52 ± 0.04^aB^	3.23 ± 0.14^aA^	3.51 ± 0.07^aB^	3.94 ± 0.05^bC^
	PL	3.59 ± 0.06^aB^	3.23 ± 0.05^aA^	3.50 ± 0.17^aB^	3.87 ± 0.07^bC^
	PB‐L	3.77 ± 0.07^bA^	3.59 ± 0.04^cA^	3.79 ± 0.06^bA^	4.07 ± 0.1^cB^
	PB‐S	4.68 ± 0.08^eB^	4.19 ± 0.07^dA^	4.20 ± 0.1^cA^	4.21 ± 0.07^cA^
	PL‐S	4.31 ± 0.03^cA^	4.33 ± 0.08^eA^	4.60 ± 0.17^dB^	4.51 ± 0.15^dB^
	PBL‐S	4.23 ± 0.05^cB^	4.20 ± 0.11^dB^	4.21 ± 0.05^cB^	4.48 ± 0.04^dC^
Acidity (%)	C	1.65 ± 0.09^dA^	2.1 ± 0.03^dB^	1.94 ± 0.08^eB^	2.05 ± 0.1^fB^
	CS	1.57 ± 0.04^dB^	0.95 ± 0.07^aA^	0.90 ± 0.0^aA^	0.90 ± 0.04^aA^
	PB	1.85 ± 0.1^eB^	1.93 ± 0.14^dC^	1.96 ± 0.05^eC^	1.65 ± 0.03^eA^
	PL	1.85 ± 0.07^eAB^	1.94 ± 0.07^dCB^	1.65 ± 0.04^dA^	1.80 ± 0.11^fAB^
	PB‐L	1.1 ± 0.03^bA^	1.25 ± 0.06^cB^	1.34 ± 0.08^cB^	1.36 ± 0.09^dB^
	PB‐S	0.80 ± 0.06^aA^	1.25 ± 0.08^cB^	1.15 ± 0.06^bB^	1.15 ± 0.02^cB^
	PL‐S	1.20 ± 0.03^cbB^	0.94 ± 0.04^aA^	1.05 ± 0.1^bA^	1.01 ± 0.05^bA^
	PBL‐S	1.15 ± 0.04^bA^	1.05 ± 0.06^bA^	1.01 ± 0.09^bA^	1.30 ± 0.08^dB^
Syneresis (%)	C	63.93 ± 3.14^aA^	67.13 ± 5.3^baA^	58.94 ± 2.58^aA^	65.05 ± 6.25^aA^
	CS	73.02 ± 2.15^cB^	70.03 ± 4.17^baAB^	65.06 ± 1.17^baA^	62.03 ± 2.27^aA^
	PB	67.13 ± 3.34^aA^	67.03 ± 7.14^baA^	63.16 ± 3.15^aA^	65.08 ± 3.35^aA^
	PL	65.12 ± 1.92^aA^	66.14 ± 2.57^baA^	66.20 ± 4.04^baA^	66.00 ± 2.05^aA^
	PB‐L	69.82 ± 3.03^cbaA^	68.93 ± 2.09^baA^	67.24 ± 6.00^baA^	66.16 ± 4.14^aA^
	PB‐S	63.03 ± 2.06^aA^	64.13 ± 0.08^aA^	63.09 ± 2.06^aA^	64.56 ± 1.26^aA^
	PL‐S	76.02 ± 2.95^cA^	74.04 ± 6.67^baA^	75.18 ± 5.01^baA^	74.01 ± 9.05^baA^
	PBL‐S	66.17 ± 1.54^aB^	60.09 ± 3.16^aA^	60.05 ± 1.19^aA^	66.06 ± 2.28^aB^

*Note*: In each column, different lowercase letters indicate significant differences among treatments, and different uppercase letters in the same row indicate significant differences among the samples during the storage (*p* < .05).

Abbreviations: C, Probiotic‐free and shallot‐free yogurt, starter only; CS, yogurt with shallots; PB, Bifidobacterium probiotic yogurt; PB‐L, Bifidobacterium and Lactobacillus probiotic yogurt; PBL‐S, Bifidobacterium and Lactobacillus with shallot yogurt; PB‐S, Bifidobacterium and shallot yogurt; PL, Lactobacillus probiotic yogurt; PL‐S, Lactobacillus and shallot yogurt.

When adding different compounds to yogurt, attention should be paid to the possibility of creating changes in syneresis. The values of syneresis were comprised between 76% in PL‐S (day 1) and 58% in control samples (day 14). The lowest amount of syneresis was obtained in the PB‐S treatment on day 1 of the experiments, significantly different from the highest amount in PL‐S (*p* < .05). During the experiment, the amount of syneresis in the PL‐S samples decreased less, and on the last day (day 21), the amount of syneresis in this treatment was significantly higher than in the rest of the samples (*p* < .05). The presence of probiotic bacteria and shallot in the yogurt in Ramezani et al.'s study increased syneresis, which is consistent with the results of our study that the presence of *Lactobacillus* and shallot caused a high level of syneresis (Ramezani et al., 2019).

The presence of probiotic bacteria and shallot has an effect on the physicochemical properties of the yogurt. The pH of the produced yogurts is one of the characteristics that changed under the influence of the synergistic properties of the produced samples. The presence of probiotic bacteria at the incubation temperature increases the production of lactic acid, and this increase in production causes significant changes in the pH of the product on certain days, especially in PB and PL treatments, according to the results of Singh et al. (2011). The cold environment does not completely stop fermentation but significantly reduces the activity of lactic acid bacteria and other microorganisms involved in the fermentation process. In one study, the addition of shallot along with inulin was shown to increase the rate of pH reduction in yogurt samples (Farahbaksh & Roufegarinejad, 2021). The presence of shallot had no significant effect on the pH changes, except for one treatment (PBL‐S) in the first week. Research on yogurt mixed with *Allium iranicum* powder showed that as the concentration of the powder increased, the acidification rate of the samples slowed down during storage (Pirsa et al., 2019).

### 3.2 Microbial analysis

According to the results, coliforms, mold, and yeasts were not detected in any of the treatments containing shallot and probiotic bacteria after 21 days of cold storage; these results are in agreement with and supported by other studies (Elkot et al., 2022; Shahein et al., 2022). Only in the control sample were observed mold and yeast, which ranged from 1.15 to 2.53 log cfu g^−1^ throughout the storage days.

In many studies, the antimicrobial activity of shallot and its isolated compounds has been demonstrated both in vitro and in vivo, as well as their preservation properties. Allium family compounds have been shown to possess inhibitory activity against a broad range of microorganisms, including bacteria, fungi, viruses, and parasites. Allium‐derived antimicrobial compounds inhibit microorganisms by interacting with sulfhydryl (SH) groups of target proteins (Moldovan et al., 2022).

**FIGURE 1:**
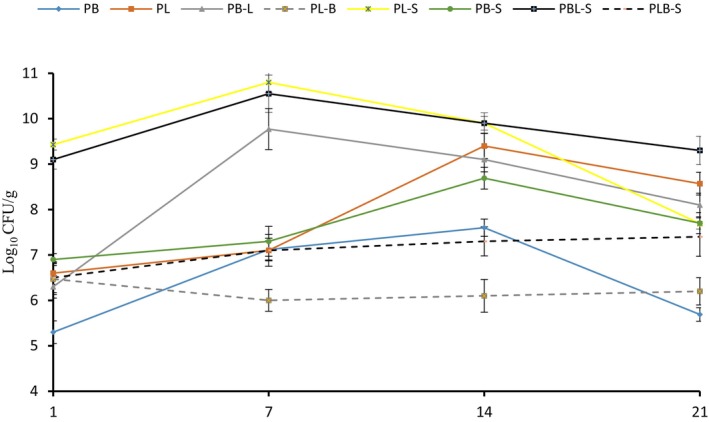
The survival rate of probiotic bacteria during storage time. PB, Bifidobacterium probiotic yogurt; PB‐L, Bifidobacterium and Lactobacillus probiotic yogurt, Lactobacillus count; PBL‐S, Bifidobacterium and Lactobacillus and shallot probiotic yogurt, Lactobacillus count; PBL‐S, Bifidobacterium and Lactobacillus with shallot yogurt; PB‐S, Bifidobacterium and shallot probiotic yogurt; PB‐S, Bifidobacterium and shallot yogurt; PL, Lactobacillus probiotic yogurt; PL‐B, Bifidobacterium and Lactobacillus probiotic yogurt, Bifidobacterium count; PLB‐S, Bifidobacterium and Lactobacillus and shallot probiotic yogurt, and Bifidobacterium count; PL‐S, Lactobacillus and shallot probiotic yogurt; PL‐S, Lactobacillus and shallot yogurt.

The survival of probiotic bacteria added to the treatments is shown in Figure 1. According to the results, the survival rate of probiotic bacteria in all samples increased up to 7 days. This increase continued significantly for the count of Lactobacillus in the probiotic yogurt (PL) and Bifidobacterium in shallot yogurt (PB‐S) until day 14 (*p* < .05). On day 21, the highest survival was recorded in the sample PBL‐S (Bifidobacterium and Lactobacillus and shallot yogurt). A decreasing trend in treatment was observed from day 14 to day 21. However, a one‐to‐one comparison between treatments containing shallot and those without shallot shows that the counts of Lactobacillus and Bifidobacterium bacteria are high on all storage days in treatments containing shallot. Many studies show that the number of probiotic bacteria decreases significantly during refrigerated storage, especially in acidic environments. In fermented products such as yogurt, the low pH and high acidity of the product on the one hand, and the cultivation of nonprobiotic lactic acid bacteria with probiotics on the other hand, cause faster death of probiotics during storage, especially in the final stages. The reason for the decrease in the number of probiotic bacteria in the third week of storage is consistent with the results of this study (Saarela et al., 2000).

Ranadheera et al. (2012) evaluated the viability of probiotics in white and stirred fruit yogurt made from goat milk and showed that in all types of yogurts during storage, *Bifidobacterium* BB‐12 was reported to be higher than the minimum therapeutic level but alive *Lactobacillus* in yogurts was less than 10^6^ CFU/g, resulting in poor growth and subsequent poor shelf‐life, most likely due to nutrient competition, which is consistent with the results of the present study (Ranadheera et al., 2012).

In a recent study, the results showed that the addition of a certain percentage of cinnamon to the yogurt reduced the number of viable lactic acid bacteria (Jiménez‐Redondo et al., 2022). In the case of *Lactobacillus*, however, shallots increase the number of viable bacteria, which shows that if shallot is used in the right amount and setting, its antibacterial effects can be reduced and its synbiotic effects can be increased. Kariyawasam et al. used a novel strain of *Lactobacillus brevis* along with fructo‐oligosaccharides (FOS) in an enriched yogurt stored at 4°C for 21 days. The results of their study showed that the increase in the number of lactic acid bacteria in the presence of FOS and probiotics is very high, especially in the first week (Kariyawasam et al., 2021). Ashrafian et al. (2022) investigated the effect of ethanolic and aqueous extracts of shallot on the survival of yogurt starters (*Streptococcus thermophilus* and *Lactobacillus bulgaricus*). The results revealed that the yogurt starter bacteria in the presence of shallot extracts were alive and active until day 14 of refrigeration.

According to some authors (Adebola et al., 2014; Kariyawasam et al., 2021), the presence of synbiotic compounds can be a source of energy and a stimulus for increased survival of lactic acid bacteria. The most important of these phytochemicals are phenolic compounds, organic sulfur compounds, polysaccharides and saponins. Also, shallot has a high content of the flavanolic compound quercetin, in its conjugated form with saccharides (Adebola et al., 2014; Major et al., 2022). The results of many researches have shown that the use of plant compounds in certain quantities not only does not have an inhibitory effect on the starter and probiotic bacteria but can also increase their growth (Altuntas & Korukluoglu, 2019).

### 3.3 Pathogen growth inhibition

**FIGURE 2:**
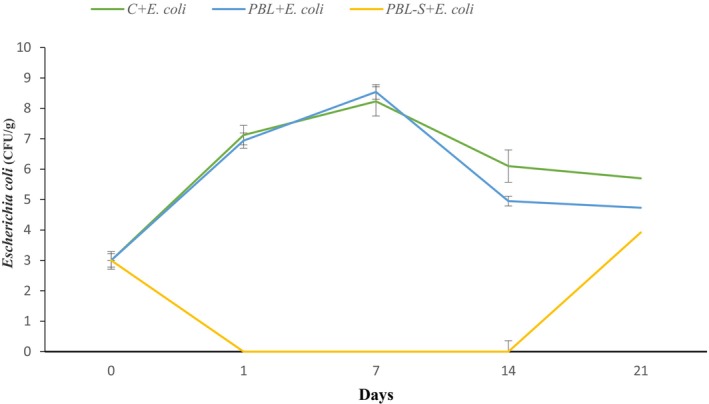
Changes in *Escherichia coli* count in different yogurt treatments during storage. Bifidobacterium and Lactobacillus probiotic yogurt containing shallot and *E. coli* (PBL‐S + *E. coli*), and Bifidobacterium and Lactobacillus probiotic yogurt containing *E. coli* without shallot (PBL + *E. coli*).

The changes in the bacterial load of *Escherichia coli* in the different yogurt treatments during storage are shown in Figure 2. According to the results, the treatment containing probiotic bacteria and shallot had a very good inhibitory effect on *Escherichia coli* bacteria. No growth of *Escherichia coli* was observed until day 14.

Microbial contamination of food and water sources has been one of the most important factors in the prevalence of diseases caused by *Escherichia coli* (Pajohi Alamoti et al., 2022). In this study, *Escherichia coli* H7:O157 (1533 ATCC) was added to milk as a foodborne pathogen in the amount of 10^3^ CFU mL^−1^, and then yogurt was prepared and stored for 21 days. The results show that in the control sample of yogurt, *Escherichia coli* bacteria grew well between days 1 and 7, but due to the low pH and acidic conditions of the yogurt, the growth rate of *Escherichia coli* decreased from days 7 to 21. In the case of yogurt containing *Lactobacillus acidophilus* and *Bifidobacterium bifidum* without shallots, the amount of *Escherichia coli* increased between day 1 and day 7 and decreased on day 14, and then remained constant until day 21. In the case of the sample containing shallots, however, no growth was observed between days 1 and 14, indicating the antimicrobial properties of shallots, but on day 21, the growth rate of *Escherichia coli* increased. This shows a significant difference in the level *p* < .05.

**FIGURE 3:**
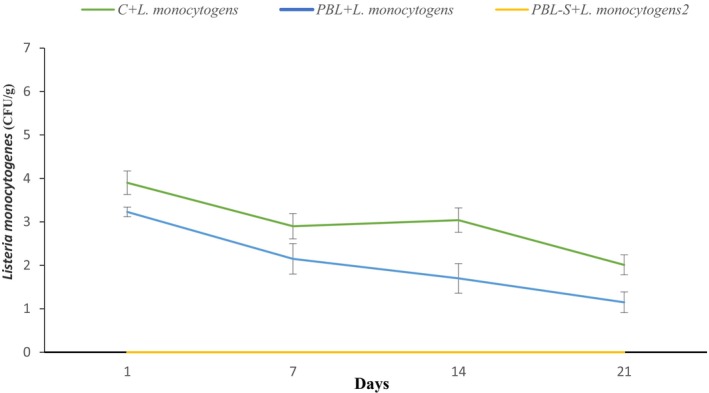
Changes in *Listeria monocytogenes* count in different yogurt treatments during storage period. Bifidobacterium and Lactobacillus probiotic yogurt containing shallot and *Listeria monocytogenes* (PBL‐S + *L. monocytogens*), and Bifidobacterium and Lactobacillus probiotic yogurt containing *L. monocytogens* without shallot (PBL + *L. monocytogens*).

The changes in *Listeria monocytogenes* in the different yogurt treatments during sample storage are shown in Figure 3. Treatment with probiotic bacteria and shallot completely inhibited the growth of *Listeria monocytogenes* during the entire storage period (21 days).

*Listeria monocytogenes* is one of the most important foodborne pathogens and there are many reports of the prevalence of gastroenteritis caused by this bacterium in humans (Al‐Nabulsi et al., 2016). In Figure 3, in the control sample in the refrigerator between days 1–7, the amount of *Listeria monocytogenes* decreased between days 7–14 and increased until the last day during 21 days of storage. The lowest counts were recorded on the 21st day. The decrease on the 21st day has a significant relationship because as the pH of the yogurt decreases, the bioavailability of *Listeria monocytogenes* decreases. Days 1–7 decreased significantly, but between days 7 and 21, the growth rate of *Listeria monocytogenes* decreased slowly due to the presence of lactic acid bacteria. In the samples of probiotic yogurt with shallots (PBL‐S), no growth was observed at all during the 21 days of the experiment, which shows a significant difference in level (*p* ≤ .05).

In vitro studies showed that phytochemicals in spices significantly enhanced the growth of probiotics and inhibited pathogens in yogurt. However, it is important to note that further research is needed to confirm these findings and understand the specific mechanisms involved (Be et al., 2009; Sutherland et al., 2009). *Escherichia coli* is one of the most important foodborne pathogens. Various methods exist to treat this pathogen, but many of these methods cause changes in the sensory characteristics of food (Pajohi Alamoti et al., 2022). One of the new methods that can inhibit the growth and survival of this pathogen and also have positive effects on the sensory properties of food is the use of probiotics (Puligundla & Lim, 2022). In a study, the presence of essential oil together with probiotic bacteria in cheese had high synergistic effects against *E. coli*, in accordance with the findings of the present study (Taina Diniz‐Silva et al., 2020). Fathy et al. used citrus peels to prevent the growth of food pathogens in yogurt and their results showed that the use of this compound can inhibit growth of *E. coli* compared to the control group, which was consistent with the results of our study (Fathy et al., 2022).

*Listeria monocytogenes* is an important foodborne pathogen, especially in dairy products and ready‐to‐eat foods. The use of lactic acid bacteria and probiotics in controlling *L. monocytogenes* in foods, especially ready‐to‐eat foods, is of great interest (Webb et al., 2022). Prezzi et al. (2020) investigated the effect *of Lactobacillus rhamnosus* in inhibiting *Listeria monocytogenes* and *Staphylococcus aureus* in Minas Frescal cheese. Similar to the results obtained in our study, *Lactobacillus rhamnosus* was able to inhibit the growth of *Listeria monocytogenes*, but had no significant effect on the growth of *Staphylococcus aureus* (Prezzi et al., 2020).

In another study, China et al. (2012) showed that the polyphenol extract of *Sesbania grandiflora* has an inhibitory effect on pathogens and stimulates the growth of probiotic bacteria (China et al., 2012). The combination of probiotic bacteria and prebiotic, in the form of synbiotic products, can cause the release of antibacterial substances such as bacteriocin, which can limit the growth of pathogenic microorganisms. Shallots are known for their high content of phenolics, especially the flavanol quercetin, in its conjugated form with saccharides; this can also be another reason for the reduction in pathogens in the present research.

### 3.4 Sensory evaluation

The results of sensory evaluation of the treatments are shown in Table 3. The results revealed that there were no significant differences in texture, color, and general appearance of yogurts, and no negative impact on these parameters was observed. This means that it can be added to plain unflavored yogurt or probiotic yogurt. In terms of taste, PB‐L, PB‐S, PL‐S, and PBL‐S treatments had a significantly higher score (*p* < .05). Although adding shallot can improve the taste of yogurt, it should be kept in mind that the amount of shallot added should be sufficient to appeal to consumers. Regarding yogurt, the texture and firmness of the product are very important from the consumer's point of view (Shori et al., 2022).

**TABLE 3:** Sensory properties of treated yogurt samples.

	Taste	Texture	Color	General appearance
C	2.9 ± 0.56^a^	3.5 ± 0.52^a^	3.6 ± 0.51^a^	3.6 ± 0.51^a^
CS	2.9 ± 0.73^a^	3.0 ± 0.66^a^	3.4 ± 0.51^a^	3.2 ± 0.51^a^
PB	3.0 ± 0.47^a^	3.9 ± 0.31^a^	3.0 ± 0.0^a^	3.8 ± 0.42^a^
PL	2.9 ± 0.56^a^	3.6 ± 0.51^a^	3.0 ± 0.47^a^	3.6 ± 0.51^a^
PB‐L	3.5 ± 0.52^b^	3.5 ± 0.52^a^	3.2 ± 0.42^a^	3.1 ± 0.31^a^
PB‐S	3.7 ± 0.48^b^	3.7 ± 0.48^a^	3.8 ± 0.42^a^	3.0 ± 0.47^a^
PL‐S	3.9 ± 0.48^b^	3.5 ± 0.56^a^	3.6 ± 0.51^a^	3.5 ± 0.56^a^
PBL‐S	3.7 ± 0.48^b^	3.2 ± 0.42^a^	3.1 ± 0.31^a^	3.2 ± 0.42^a^

*Note*: The mean values followed by the same letters in the column are not significantly different (*p* < .05).

Abbreviations: C, Probiotic‐free and shallot‐free yogurt, starter only; CS, yogurt with shallots; PB, Bifidobacterium probiotic yogurt; PB‐L, Bifidobacterium and Lactobacillus probiotic yogurt; PBL‐S, Bifidobacterium and Lactobacillus with shallot yogurt; PB‐S, Bifidobacterium and shallot yogurt; PL, Lactobacillus probiotic yogurt; PL‐S, Lactobacillus and shallot yogurt.

In a study, the addition of several prebiotics and commercial fiber (inulin, HAM‐RS, lactitol, lactulose, beta‐glucan, and maltodextrin) at two levels (1.5% and 3.0%) resulted in a lower sensory acceptance compared to the control group (Heydari et al., 2011). In contrast, several studies have shown that the addition of probiotic bacteria increases the sensory score of yogurts. In the results of Gündoğdu et al. (2009), on the addition of garlic and the evaluation of the characteristics and shelf life of stirred yogurts, it was stated that the highest overall score was obtained in yogurt treated with 1% garlic. Sánchez‐Vega (2013) in a study on the production of yogurt with garlic extract, ginger extract, and 0.05% turmeric reported that the sample containing ginger scored 84% (Sánchez‐Vega, 2013). According to results of one study, yogurt enriched with probiotic bacteria and fructo‐oligosaccharide did not cause any significant change in sensory properties (Kariyawasam et al., 2021), but probiotic bacteria could improve the sensory properties of yogurt, which can be very important in attracting the consumer's opinion.

## 4 CONCLUSION

The results of this study showed that the use of probiotic bacteria (either alone or in combination with shallot) can increase the microbial and physicochemical quality of yogurt. Furthermore, the supplementation of probiotic and prebiotic compounds not only did not negatively affect the sensory acceptance of the product with shallots but also significantly improved the taste of the yogurt. Additionally, the viability of probiotic bacteria (>7 log cfu/mL) was also positively improved by the incorporation of shallot in yogurt during 21 days of cold storage. Then, synbiotic yogurt containing probiotic bacteria along with shallot was also able to show significant inhibitory activity on *Escherichia coli* and *Listeria monocytogenes* bacteria as foodborne pathogens.

## AUTHOR CONTRIBUTIONS

Farahnaz Vahdat: Software, investigation, and data curation; Tooraj Mehdizadeh: Conceptualization, methodology, software, validation, formal analysis, investigation, resources, data curation, writing–review and editing, visualization, supervision, project administration, and funding acquisition; Hamidreza Kazemeini: Supervision and project administration; Anna Reale: Software, writing–original draft preparation, and visualization; Ata Kaboudari: Methodology, investigation, data curation, writing – original draft preparation, writing–review and editing, and visualization.

## FUNDING INFORMATION

The funding was provided by Amol University of Special Modern Technologies, Faculty of Veterinary Medicine, and research grant for postgraduate theses.

## CONFLICT OF INTEREST STATEMENT

There is no conflict of interest.

## Data Availability

The authors declare that the data supporting the findings of this study are available within the paper.
